# OrChem - An open source chemistry search engine for Oracle^®^

**DOI:** 10.1186/1758-2946-1-17

**Published:** 2009-10-22

**Authors:** Mark Rijnbeek, Christoph Steinbeck

**Affiliations:** 1 Cheminformatics and Metabolism, European Bioinformatics Institute (EBI), Wellcome Trust Genome Campus, Cambridge, UK

## Abstract

**Background:**

Registration, indexing and searching of chemical structures in relational databases is one of the core areas of cheminformatics. However, little detail has been published on the inner workings of search engines and their development has been mostly closed-source. We decided to develop an open source chemistry extension for Oracle, the de facto database platform in the commercial world.

**Results:**

Here we present OrChem, an extension for the Oracle 11G database that adds registration and indexing of chemical structures to support fast substructure and similarity searching. The cheminformatics functionality is provided by the Chemistry Development Kit. OrChem provides similarity searching with response times in the order of seconds for databases with millions of compounds, depending on a given similarity cut-off. For substructure searching, it can make use of multiple processor cores on today's powerful database servers to provide fast response times in equally large data sets.

**Availability:**

OrChem is free software and can be redistributed and/or modified under the terms of the GNU Lesser General Public License as published by the Free Software Foundation. All software is available via http://orchem.sourceforge.net.

## Introduction

**Figure 1:**
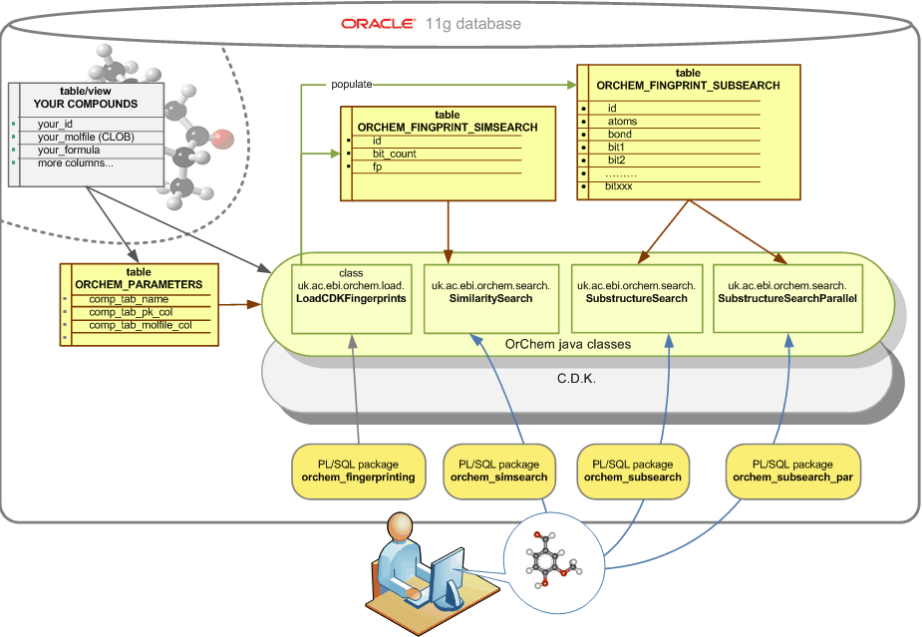
**OrChem overview**.

Registration, indexing and searching of chemical structures in relational databases is one of the core areas of cheminformatics [1,2]. Research on the topic goes back to the 1960s and probably before that [3]. However, little detail has been published on the inner workings of search engines and developments have been mostly closed-source. This has led to the situation that despite more than thirty years of research and publications very few open reference code is available for use and study. The cheminformatics open source community [4] has been working since the mid 1990s to overcome this problematic situation. Our group has contributed to this by creating and developing the Chemistry Development Kit (CDK) [5,6], now co-developed with collaborators world-wide as well as NMRShiftDB, an NMR database which provides a MySQL-based open source system for the registration and searching of chemical compounds in a relational database [7,8]. In the meantime, three projects dedicated to the development of chemical registry and search capabilities for PostgreSQL (project PGChem) [9], MySQL (project MyChem) [10] and Oracle (project OrChem) [11] have been established under the umbrella of the ChemiSQL-Project (pronounced "chemiscule") [12]. Here we report about our development of OrChem, an open source software package which adds registration and chemical search capabilities to the Oracle 11G database system. OrChem is a mix of PL/SQL and Java that executes inside the database. Figure 1 gives an overview of OrChem's main components, showing how the user interacts with OrChem via PL/SQL packages that call out to so called "Java Stored Procedures" [13]. Starting with Oracle 11 g there is a just-in-time (JIT) compiler for the Oracle JVM environment. A just-in-time (JIT) compiler is a program that converts Java bytecode into machine language instructions which makes Java run much faster than when the bytecode is executed by an interpreter.

OrChem is built on top of the Chemistry Development Kit (CDK) [5,6] and depends on this Java library in numerous ways. For example, compounds are represented internally as CDK molecule objects, the CDK's I/O package is used to retrieve compound data, and its subgraph isomorphism algorithms are used for substructure validation. OrChem adds its own Java layer on top of the CDK to implement fast database storage and retrieval. With the CDK loaded into Oracle, a large cheminformatics library becomes readily available to PL/SQL. With little effort developers can build database functions around the CDK and so quickly implement chemistry extensions for Oracle. OrChem works in the same way, using the CDK where possible.

## Fingerprinting

OrChem uses chemical fingerprints to find compounds by substructure or similarity criteria. Fingerprints are bitsets in which each bit indicates the presence or absence of a particular chemical aspect. During a similarity search the fingerprints are used to calculate a Tanimoto measure [14]. A Tanimoto similarity measure between two binary fingerprints is defined by the ratio of the number of common bits set to one to the total number of bits set to one in the two fingerprints [15]. For substructure searching the fingerprint has a different function: it is used to screen possible candidates before a computationally more expensive isomorphism test.

For both substructure and similarity searching OrChem uses the same fingerprint that currently measures around 800 bits in size and uses both structural keys and hashed values. With structural keys each position in the bit string corresponds to a pre-defined structure or molecular feature. A hashed fingerprint is produced by generating all possible linear paths of connected molecules containing between one and a defined number of atoms, and projecting the hash values of the resulting strings onto the small set of bits in the fingerprint in a deterministic manner [16].

In the OrChem fingerprint the first approximately one hundred bits are reserved for hashing three-atom SMILES strings. If a compound contains for example "C-C:S" then bit number 20 will be set, as the calculated hash value is 20 for this particular example. A hash value is calculated consistently for each pattern encountered. We only take three-atom SMILES into account because these yield a distinct yet relatively small amount of possible combinations, all of which can be properly hashed into the limited amount of bits reserved for the hashed key in the fingerprint.

Hashing in general decreases the accuracy of Tanimoto scoring because different chemical aspects will hash to the same bit position. However the benefit of hashing lies in flagging relatively rare patterns. Rare SMILES patterns would normally not be part of a structural key because assigning an explicit bit would be wasteful, but by hashing all size three patterns into a range of bit positions the infrequent patterns still leave their mark and this can speed up substructure searches. The OrChem fingerprint was at first only structural and structure searches for an unusual SMILES pattern like "O:C:O" saw many compounds being screened in vain. The current fingerprint hashes "O:C:O" to a bit position and this helps to narrow down the set of candidates significantly.

Coming after the hashed part, the structural key in the OrChem fingerprint starts around bit position one hundred. The structural key was initially based on the PubChem fingerprint [17,18] but the current version differs from it in various ways. OrChem for instance flags specific SMILES patterns that have proved discriminating for searching compounds like those in ChEMBL [19]. Numerous bits are reserved to capture ring aspects: clusters of rings are detected and aspects of these clusters are fingerprinted, like the occurrence of a ring size three attached to a ring size five. With regards to the common rings of size five and six, OrChem creates textual descriptors for every connected set of three of these encountered and incorporates this information into the fingerprint. Example textual descriptors are "5DLM" and or "6SHH", the meaning being as follows:

• Character 1: {"6","5"} indicates if the ring triplet contains strictly 6-size rings or otherwise (up to three) 5-size rings

• Character 2: {"S","D"} indicates bond nature, S means single bonds only, D double bonds (possibly aromatic) also present

• Character 3: {"L","H"} indicates connectivity, H means some atom participates in all 3 rings (tightly coupled)

• Character 4: {"L","M","H"} indicates shared bonds between rings in the triplet: Low = 0,1,2 Med = 3,4 High = 5..n

The aim of these descriptors is to characterize particular constellations of common sized rings, to identify structures that look normal purely based on the ring sizes but may be in fact rare due to the way the rings are connected together in a cluster.

Future OrChem releases may see the structural key further extended for improvement of specific search types. For now, in summary the fingerprint captures the following aspects:

• hashed three-atom SMILES strings

• element counts

• atom pairs

• atom nearest neighbours

• common SMILES patterns

• individual rings: size, aromaticity, elements

• ring clusters: cluster size, what ring sizes in the cluster

• 5/6 size ring triplets: how are rings connected, how many atoms and bonds are shared

• long carbon trails: longer series of (non aromatic) carbon molecules

• infrequent SMILES patterns (grouped per bit)

**Figure 2:**
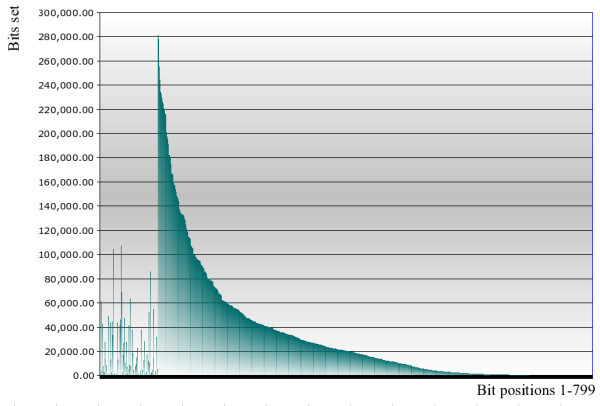
**OrChem fingerprint distribution in Chembl database**.

Figure 2 shows the frequency distribution of OrChem fingerprint bits for all compounds in the ChEMBL database. The dual nature of the fingerprint is clearly visible: the first part shows a randomly distributed hashed key, followed by the ordered structural key. Ordering the structural key by occurrence of bits makes sense because the bits get indexed using composite B-tree indexes. Clustering rare bits at the tail of the fingerprint makes the corresponding indexes small and provide an obvious pick for the Oracle optimizer.

## Similarity searching

With the fingerprint in place OrChem can perform fast similarity searches. The algorithm for similarity searching is taken from a paper by S. Joshua Swamidass and Pierre Baldi [15].

The algorithm proves to work well and allows the search to break out when a minimal given similarity is reached, whilst completeness of the output is guaranteed. For Orchem the similarity search has been implemented as a Java stored procedure that returns an array of results. The current function accepts SMILES and Molfiles for a query and a cut-off value between 0 and 1 to indicate minimum required similarity. Optional arguments allow the user to indicate a cap to indicate the maximum number of results required, and whether or not to display debugging information. The select statement below shows a query example to find compounds with a similarity of seventy percent or more to the given SMILES string:

select *

from table(orchem_simsearch.search

('CCCCCC[C@H]1CC[C@H]2CCCC[C@]12C','SMILES',0.7))}

At the center of the similarity search is a table called orchem_fingprint_simsearch, pictured in Table 1. The implemented algorithm uses the bitmap indexed column bit_count to first inspect compounds for which the number of bits set to one is the same as that of query molecule. After that it works its way through compounds with a bit count close to that of the query until it is done, that is until the minimum similarity has been reached or until the result set size satisfies the user. Column fp in the similarity table stores a byte array representation of the fingerprint. This raw data column allows the similarity search to quickly read bytes from the database, convert these to Java bitsets and use those for bitwise comparisons to obtain a Tanimoto similarity score.

**Table 1:** Database table with fingerprints optimized for similarity searching

TABLE orchem_fingprint_simsearch	
id	VARCHAR2(32)

bit_count	NUMBER(4)

fp	RAW(100)

## Substructure searching

### Prescreening

Chemical fingerprints can be used to quickly pre-screen candidates likely to contain a given query as a substructure [20]. Due to the degeneracy of bits in a fingerprint, this leads to false positives. The screened set of candidates thus needs further inspection by an isomorphism algorithm to detect if the substructure is truly present or not. A substructure search is therefore a two step process - ideally the first step uses the fingerprint to screen accurately so that the computationally more expensive second step will have a high ratio of positive verifications. The other way around, if the first step is not efficient, the second step will have to inspect too many compounds that don't contain the substructure at all.

The screening process is in essence a bitwise comparison between the two fingerprints of a query and a candidate structure. Any bit set to true in the query fingerprint must be set to true in a candidate fingerprint. OrChem implements this comparison using a dedicated database table that is partly listed in Table 2. Database table orchem_fingprint_subsearch has a separate column for each bit in the fingerprint; this is different from the table for similarity searching that has a single raw data column to store an entire fingerprint. The reason behind this difference is that for similarity searching the bitwise operation is done in Java with binary bitsets, whereas the substructure screening uses the separate bit columns to construct a dynamic SQL clause instead. This bit-column based "where" clause instructs the database to select compounds for which bits in the fingerprint set to "1" include those set to "1" in the query structure. For instance a substructure search for "P = O" will have OrChem create a SQL clause using the three bits that are set in the fingerprint of "P = O". The pre-screening query will resemble:

**Table 2:** Database table with fingerprints optimized for substructure searching

TABLE orchem_fingprint_subsearch	
id	VARCHAR2(80)

atoms	VARCHAR2(4000)

bonds	VARCHAR2(4000)

bit0	VARCHAR2(1)

bit1	VARCHAR2(1)

*etc...*	

bit799	VARCHAR2(1)

select ...

from orchem_fingprint_subsearch

where bit472='1' and bit477='1' and bit480='1'}

The meaning of the bits is not relevant here, the point is that the OrChem prescreening process is essentially done through a SQL clause filtering for indexed bit columns that correspond to bits set in the fingerprint of the query. The bitwise comparison is thus replaced entirely by a single SQL statement with no need for computationally expensive Java.

### Exact search for subgraph isomorphism

For each compound that passes the prescreening process, a graph matching algorithm needs to establish whether the query indeed occurs as a substructure or not. Molecules can easily be interpreted as graphs with atoms being nodes and bonds being edges. The graph matching in Orchem is done by a CDK Java implementation of the VF2 algorithm [21]. VF2 is a graph matching algorithm that has been shown to perform better than the Ullmann algorithm for small graphs, and much better than Ullmann for large graphs. Compared to the original VF algorithm, VF2 lowers the memory requirements from O(n^2^) to O(n) with respect to the number of nodes in the graphs.

VF2 is a fast backtracking algorithm that tries to match each node in a query graph to a node in a target graph. OrChem further improves VF2 performance by reorganizing the query graph beforehand by sorting the nodes (atoms) and edges (bonds). A primary sort is done based on the uniqueness of elements and a secondary sort on bond connectivity. Essentially the sort moves the rare nodes up to be matched first: if the query structure is C16H14N2O9S then it is best to let VF2 try to match sulfur first, then the nitrogens, then the oxygens et cetera. The secondary sort on bond connectivity can be useful if the query does not contain distinctive atoms but has distinctive structures such as ring groups in which some atoms are more connected than others.

The performance of the Java VF2 implementation mainly depends on the complexity and characteristics of the molecular input graphs. The algorithm may need to get recursively deep, or may need to explore many possible mappings, making the graph matching algorithm the computationally expensive part of the substructure search.

### Query example

The developer can submit an OrChem substructure search through package orchem_subsearch:

select *

from table (orchem_subsearch.search

('S:C:C','SMILES',1000))

The example shows a substructure search for SMILES "S:C:C" with a break out after 1000 results through an optional argument that emulates the ROWNUM column. Under the hood the SMILES string will be fingerprinted, a prescreen query will be constructed based on it and then executed with the Oracle Optimizer picking the most suitable index available for the bit columns. Each candidate will be verified using VF2 and only valid superstructures will be returned. The substructure search function is *pipelined*, so rows are returned iteratively as they are produced without having to wait for the entire collection to be constructed. This allows developers to build web interfaces refreshing at a constant interval while presenting the results returned so far to the user.

## Performance

### General

OrChem has been tested extensively on a release of the ChEMBL database (former Starlite) with around 420,000 compounds. Additional tests were done on a random PubChem data sample of 3.5 million compounds in order to assess performance on a larger data set. All tests were done on Oracle 11.1.0.7 installed on an eight CPU quad core 32 Gb RedHat Linux server at the EBI. With regards to performance, it is good to keep in mind that the initial run of any Java stored procedure incurs a lot of overhead. Even a first call to a simple 'helloWorld()' program takes a while to complete, but following calls then respond immediately. Orchem's search performance improves after a few queries have been issued and the fingerprint data starts to accumulate in the buffer cache where data can be accessed faster than by reading from disk. The tables that support similarity and substructure searching are created with the cache option, so the more memory assigned to the SGA the better the performance will be.

### Similarity searching

**Figure 3:**
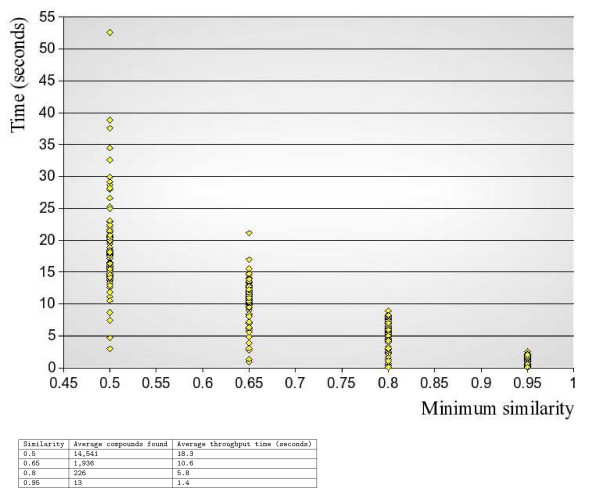
**Similarity search throughput time for different minimal Tanimoto similarity**. Explanation of graph: one hundred randomly sampled compounds were used to query for all similar compounds, repeated for different minimum Tanimoto similarities. Searches were done in the PubChem sample of 3.5 million compounds. As the similarity cutoff increases, performance goes up: finding all compounds similar to compound X with at least a Tanimoto similarity of 0.8 (= 80% similar) is faster than finding (many more) compounds that are 50% similar.

Similarity searches typically show a more consistent performance than substructure searches. To obtain a similarity score it is sufficient to compare fingerprint bitsets, and the time to complete a similarity search depends mainly on the required minimum Tanimoto similarity. Tests on ChEMBL show query throughput times in the order of split-seconds to seconds for high (*>*0.75) similarity, improving once the fingerprints start getting cached. Similar tests on the PubChem sample (3.5 million compounds) show a longer cache "warm up" time and give throughput times in the order of several seconds or longer, again mostly depending on the similarity cut-off. Figure 3 illustrates performance for searching the PubChem sample and also shows a table with average numbers with regards to query throughput time and result set sizes.

### Substructure searching

As described before, substructure searching is done as a two-step process with a prescreening step followed by a VF2 isomorphism check. In the isomorphism algorithm each screened database compound needs to be materialized as a CDK molecule to be able to compare it to the query structure. It can however be expensive to materialize thousands of molecules on the fly using database Molfiles, particularly with regards to calculation of aromaticity. Instead, OrChem stores data for each molecule in columns atoms and bonds of table orchem_fingprint_subsearch during the fingerprinting process. These columns provide a quick way to materialize a basic CDK molecule to be passed into the VF2 algorithm. The data structures used are quite straightforward, for instance with data in column atom "C O" interpreted as: "atom 0 is Carbon, atom 1 is Oxygen" and bond column "0 1 D Y" then implying "there is a bond between C (atom 0) and O (atom 1) that is double (D) and aromatic is true (Y)". In this way, CDK molecules can be generated very fast without the need for calculating any properties during the search.

### Parallel substructure searching

Oracle allows parallel execution of table functions [13] and this feature can be used to speed up Or-Chem substructure searching. A parallel table function returns a collection and is executed in a two-stage operation. First, one set of slave processes partitions the data as directed in the function's declaration; then a second set of slave scans executes the table function in parallel on the partitioned data. The following statement illustrates the concept, with *f *being the function to be run in parallel, taking a ref cursor input argument to partition the data.

select *

from table(f(cursor(select * from tab)))

Although the VF2 isomorphism algorithm is an ideal candidate for a parallel approach, going parallel is not always the best solution. OrChem can execute *non-parallel *queries relatively fast providing the query structure itself has sufficient unique features. The prescreening step for a discriminating query will normally yield a small set of database candidate compounds that can quickly be scanned with VF2. In such as case a non-parallel search can actually run faster than a parallel search because there is not much VF2 workload and no parallel overhead is incurred. But most importantly, the non-parallel search can use the regular B-tree indexes on the bit columns in table orchem_fingprint_subsearch whereas a parallel query can not.

In general, to parallelize any query Oracle must access at least one of the tables through a full table scan or an index through a range scan involving multiple partitions. In the case of Orchem this means that table orchem_fingprint_subsearch must be accessed with full table scans, but this is not always the best option. When queries are done on a database with several million compounds the indexed approach can outperform the parallel approach easily if the index can be used to quickly narrow down a small set of candidates. Furthermore the performance of a parallel query depends on a number of factors, one of them being the hardware on which the Oracle instance runs, with the more CPU cores the better. The performance also depends on the size of the database, the type of the query (specific/generic) and the volume of the result set. Tests at the EBI show that parallel queries in general perform well on the ChEMBL dataset, even if a query is very specific and an indexed approach would be quicker. This can be explained by the fact that the specific EBI hardware can run very fast full table scans on a dataset the size of ChEMBL. For larger databases this no longer holds true, as full scans become quite expensive even when run in parallel.

The invocation of a parallel substructure search needs to be done in two steps due to the underlying implementation - possibly in a later release this can be simplified. Below is an example query as done on the command line. First the substructure search is quickly set up, in this case for SMILES "C:O:C:N". A key is retrieved and this key is then used to perform the actual parallel search:

> var key number;

> exec :key :=

orchem_subsearch_par.setup('C:O:C:N','SMILES')

> select * from table

(orchem_subsearch_par.search(:key,1000)

**Figure 4:**
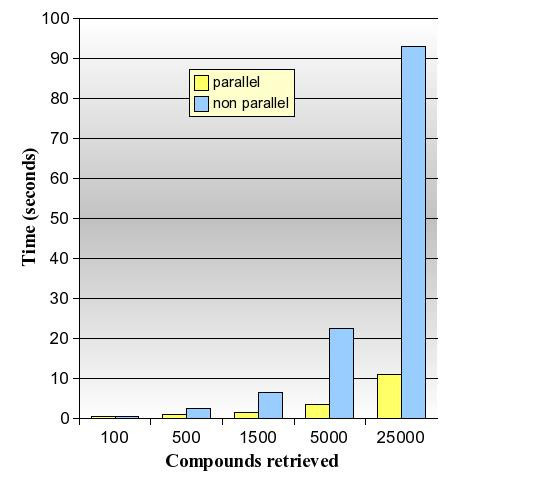
**Performance of substructure search for "C:C:C:C:N" on 3.5 million compounds**.

**Figure 5:**
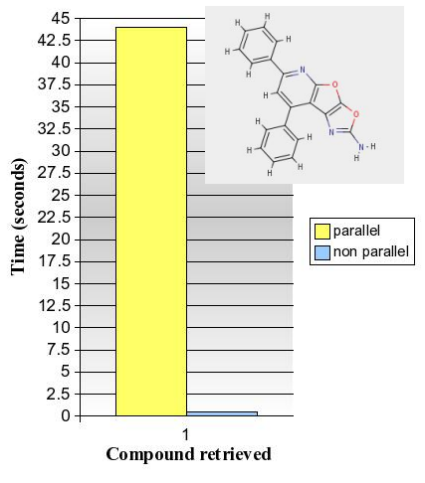
**Performance of substructure search for "C:O:C:O" on 3.5 million compounds**.

**Figure 6:**
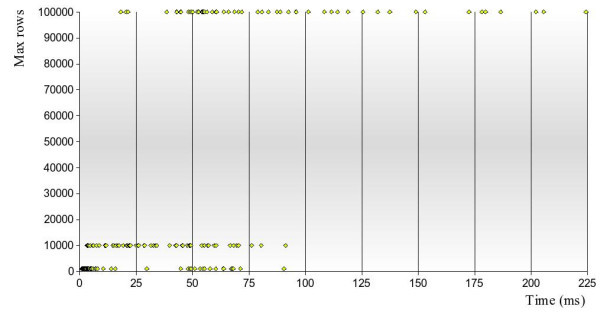
**Parallel substructure search throughput time on 3.5 million compounds**. Explanation of graph: sixty sampled substructures were used as a query for a parallel substructure search in the PubChem sample of 3.5 million compounds. The same searches were repeated with different maximum number of rows requested: 1000 (bottom), 10,000 (middle) and 100,000 (top). The graph displays the total throughput time in seconds but users can view the intermediate output generally faster as the search function spools ('pipes') results as they become available. Fastest throughput times are observed for query structures that commonly exist in compounds, resulting in high a success ratio of the VF2 isomorphism algorithm.

The parallel table function works best for general queries with high volumes of data being processed and many positive verifications being returned, the VF2 workload shared over several slave processes. This can be observed when selecting the first *n *results for a common substructure pattern like "C:C:C:C:N" with *n *set to a high value. Figure 4 shows a graph for a "C:C:C:C:N" query done against the PubChem sample: the parallel function identifies the first 5000 results in 3.5 seconds whereas the single process takes 22.5 seconds. The parallel approach clearly benefits from doing the VF2 isomorphism checks spread over multiple parallel processes, which becomes even more obvious when *n *is further increased to 25000. However to add to the picture, figure 5 shows what happens with a similar test for "C:O:C:O". This structure happens to occur only once in the entire data set, and the *non-parallel *function can use a fast index scan on the bit columns of orchem_fingprint_subsearch to quickly find three possible candidates with the right combination of fingerprint bits. One compound is then verified by VF2 to be a superstructure of "C:O:C:O" (compound is shown in the graph). The parallel approach at the other hand needs to make full table scans, albeit in parallel, and takes more than forty seconds to find the same single result. The benefit of fast parallel VF2 execution does not apply now and the elapse time is spent scanning rows that meet the rare bit combination, without being able to use any index for this. Finally in figure 6 we have a graph with throughput times for a sampled set of sixty random structures used to do a parallel substructure searching with different breakout values. The graph shows quite a spread in total throughput time, with the time to return up to 100,000 verified results varying between 20 seconds and more than three minutes, all depending on the complexity of the query, the quality of the fingerprint and the occurrence rate of the structure. Either way users can see results streamed back before completion because data is piped back as soon as it becomes available.

In conclusion the given examples show how the database content and the nature of the query determine the difference in performance between the parallel and the non-parallel substructure search. Which one is fastest depends mainly on the size of the database, the number of results requested (limited or all) and the size of the prescreened compound set. The smaller the pre-screened set is the less attractive a parallel full scan normally is - but it is hard to find exact rules. On top of that performance also depends on other factors such as the server hardware and the quality of the query's fingerprint. To make the search process more user friendly, we plan to find heuristic rules to be incorporated into a generic search function that will decide for the user whether to take a parallel approach or not.

## OrChem Installation

This paragraph briefly describes the OrChem installation process. A complete step-by-step description of the installation can be found in manuals on the project's web pages [11]. The first step is to create a new schema and necessary database elements (tables, indexes, packages) and to load required Java libraries into it. Users have to provide details of the base table containing the actual compound data, presumed to be present in another schema. Next a procedure needs to run to create a fingerprint for every compound. Each fingerprint captures the chemical aspects of a compound and is stored in the database. The amount of time it takes to create all fingerprints depends on the amount and complexity of the compounds and on the capacity of the database server. As a performance indication, at the EBI we fingerprinted over 400,000 compounds in an hour, running a parallel process with DBMS_JOB on an Oracle instance hosted on a multi-processor Linux server. Once the fingerprinting procedure has finished, installation is complete and OrChem can perform compound searches.

## OrChem future development

A number of features have been identified to be implemented in coming releases:

• The current version of OrChem expects the user's compound table to have MDL Molfiles to work with. This will be extended to include other common data formats such as CML.

• OrChem should be able to deal with R-group and SMARTS queries and to ignore bond order on request. These features have in common that wildcards should be allowed to widen the search, and the challenge will be to not only interpret these wildcards but also to keep performance acceptable.

• At present, OrChem development has not put particular emphasis on stereochemical searches. OrChem needs to be able to distinguish between stereoisomers and provide substructure search criteria related to stereoisomerism.

• More of the CDK needs to be exposed through PL/SQL API's, for example CDK functions to calculate QSAR/QSPR descriptors or chemical properties.

• The similarity search will benefit from an option to run in parallel, and more types of similarity scoring should be added to complement the current default Tanimoto scoring.

• Along the same lines we might want to experiment with different types of fingerprints.

• Pharmacophore searches could be added to take into account 3D arrangements of molecular features.

## Conclusion

We have presented OrChem, an open source extension for the Oracle 11G database platform that adds registration and indexing of chemical structures to support fast substructure and similarity searching. OrChem provides core cheminformatics functionality but is only in its first release and by no means a finished product. Developers who are interested to further extend OrChem are invited to participate through Sourceforge to make it a truly collaborative project. We also encourage users to submit suggestions and requests for functionality through the mailing list "orchem-devel@lists.sourceforge.net".

## Availability and requirements

*Project name*: OrChem

*Project home page*: http://orchem.sourceforge.net/

*Operating system*: Platform independent

*language*: Java 1.5 or higher

*Database system*: Oracle 11 g (with JRE 1.5)

*Comm. restrictions*: None

## License

OrChem is released under the terms of the GNU Lesser General Public License as published by the Free Software Foundation; either version 2.1 of the License, or (at your option) any later version.

## Competing interests

The authors declare that they have no competing interests.

## Authors' contributions

MR designed and implemented the software and drafted most of the manuscript. CS guided the development process and provided expertise on usage of the CDK. Both authors performed testing of the application, and approved the final manuscript.

## References

[B1] Hagadone T Michael S Integrating chemical structures into an extended relational database system Chemical Structures 2: The International Language of Chemistry: Proceedings of the 2nd International Conference 1995 257 269

[B2] Hagadone T Schulz M Capturing Chemical Structure Information in a Relational Database System: The Chemical Software Component Approach Journal of Chemical Information and Computer Sciences 1995 35 5 879 884

[B3] Frome J Searching Chemical Structures Journal of Chemical Documentation 1964 43 45 10.1021/c160012a009

[B4] Guha R Howard MT Hutchison GR Murray-Rust P Rzepa H Steinbeck C Wegner J Willighagen EL The Blue Obelisk - Interoperability in Chemical Informatics Journal of Chemical Information and Modelling 2005 46 3 991 998 10.1021/ci050400b PMC4878861 16711717

[B5] Steinbeck C Han YQ Kuhn S Horlacher O Luttmann E Willighagen E The Chemistry Development Kit (CDK): An open-source Java library for chemo- and bioinformatics Journal of chemical information and computer sciences 2003 43 2 493 500 12653513 10.1021/ci025584y PMC4901983

[B6] Steinbeck C Hoppe C Kuhn S Guha R Willighagen EL Recent Developments of The Chemistry Development Kit (CDK) - An Open-Source Java Library for Chemo- and Bioinformatics Current pharmaceutical design 2006 12 17 2111 2120 10.2174/138161206777585274 16796559

[B7] Steinbeck C Krause S Kuhn S NMRShiftDB - Constructing a free chemical information system with open-source components Journal of chemical information and computer sciences 2003 43 6 1733 1739 14632418 10.1021/ci0341363

[B8] Steinbeck C Kuhn S NMRShiftDB - compound identification and structure elucidation support through a free community-build web database Phytochemistry 2004 65 19 2711 2717 10.1016/j.phytochem.2004.08.027 15464159

[B9] The PGChem project 2004 http://pgfoundry.org/projects/pgchem/

[B10] The MyChem project 2004 http://mychem.sourceforge.net

[B11] The OrChem project 2004 http://orchem.sourceforge.net

[B12] The ChemiSQL umbrella project for Open Source chemical search engines 2004 http://chemdb.sourceforge.net

[B13] Oracle 11.1 database Java developer's guide (B31225-03) http://tinyurl.com/yh5kb7r

[B14] Willett P Chemical Similarity Searching Journal of Chemical Information and Modeling 1998 38 6 983 996 10.1021/ci9800211

[B15] Swamidass S Baldi P Bounds and algorithms for fast exact searches of chemical fingerprints in linear and sublinear time Journal of Chemical Information and Modeling 2007 47 2 302 317 10.1021/ci600358f 17326616 PMC2527184

[B16] Leach AR Gillet VJ An Introduction to Chemoinformatics 2007 255

[B17] Sayers E Barrett T Benson D Bryant S Database resources of the national center for biotechnology information Nucleic Acids Research 2009 37 D5 D15 10.1093/nar/gkn741 18940862 PMC2686545

[B18] Definitions of PubChem fingerprints 2004 ftp://ftp.ncbi.nlm.nih.gov/pubchem/specifications/pubchem_fingerprints.txt

[B19] The ChEMBL project at the European Bioinformatics Institute 2004 http://www.ebi.ac.uk/chembl

[B20] Barnard J Substructure searching methods: Old and new Journal of Chemical Information and Modeling 1993 33 4 532 538 10.1021/ci00014a001

[B21] Cordella L Foggia P Sansone C Vento M An improved algorithm for matching large graphs Proc of the 3rd IAPR-TC-15 International Workshop 2001

